# Recent advances in the prevention of preterm birth

**DOI:** 10.12688/f1000research.11385.1

**Published:** 2017-07-18

**Authors:** Jeff A Keelan, John P Newnham

**Affiliations:** 1 Division of Obstetrics & Gynaecology, School of Medicine, University of Western Australia King Edward Memorial Hospital, Perth, Australia

**Keywords:** Preterm birth, progesterone therapy, PTB prevention

## Abstract

Preterm birth (PTB) remains a major obstetric healthcare problem and a significant contributor to perinatal morbidity, mortality, and long-term disability. Over the past few decades, the perinatal outcomes of preterm neonates have improved markedly through research and advances in neonatal care, whereas rates of spontaneous PTB have essentially remained static. However, research into causal pathways and new diagnostic and treatment modalities is now bearing fruit and translational initiatives are beginning to impact upon PTB rates. Successful PTB prevention requires a multifaceted approach, combining public health and educational programs, lifestyle modification, access to/optimisation of obstetric healthcare, effective prediction and diagnostic modalities, and the application of effective, targeted interventions. Progress has been made in some of these areas, although there remain areas of controversy and uncertainty. Attention is now being directed to areas where greater gains can be achieved. In this mini-review, we will briefly and selectively review a range of PTB prevention strategies and initiatives where progress has been made and where exciting opportunities await exploitation, evaluation, and implementation.

## Introduction

Preterm birth (PTB) is defined as birth at >20 and <37 completed weeks of gestation. Despite decades of research, PTB remains a major obstetric healthcare problem of global significance ^1, 2^. PTB is the single major cause of death and disability in children up to 5 years of age in the developed world and the leading single cause of global perinatal mortality and morbidity ^3– 5^; approximately 15 million babies are born preterm each year worldwide, and a million of these children die ^5, 6^. Preterm infants are also at significantly greater risk of serious perinatal complications ^7– 10^. While many children born preterm lead a normal and healthy life, a significant proportion experience life-long disability and health issues ^11^. The impact on individuals, families, and society are considerable, as are the healthcare costs associated with perinatal care and life-long disability ^1, 5, 9, 12^.

In developed countries, about 30% of preterm deliveries are iatrogenic, with the remainder being spontaneous, either with intact membranes or following preterm pre-labour rupture of membranes (PPROM) ^1, 2^. Many different causes of spontaneous PTB (sPTB) have been identified, the most common of which in singleton pregnancies are likely to be intrauterine inflammation (IUI)/intrauterine infection, placental malperfusion, or other placental abnormalities ^1, 2, 7, 13, 14^. Inflammation appears to be a common mechanism underpinning multiple aetiologies ^15– 17^. While placental malperfusion pathologies are very common in singleton preterm deliveries, effective strategies to treat or prevent them—other than the administration of low-dose aspirin as anti-coagulant therapy ^18– 20^—have not been developed or tested. The causal significance of such pathologies is also uncertain. This is a relatively untapped area that deserves significant research investment ^18, 21^.

As is the case for most healthcare problems, prevention is better than cure ^22^. Successful prevention of PTB requires a multifaceted approach, combining public health and educational programs, lifestyle modification, optimisation of obstetric healthcare, effective prediction and diagnostic modalities, and the application of effective, targeted interventions ^23^. Preconception interventions in the form of weight reduction, nutritional supplementation, pharmaceutical management, and smoking cessation, etc. have the potential to make a significant impact on PTB rates ^24– 26^, although implementation and access remain significant barriers ^24, 25, 27^.

In the following paragraphs, we will provide a brief and selective overview of the recent advances in sPTB prevention, with a perspective on the opportunities for gains in the short and medium term.

## Recent advances

### The benefits and limitations of progesterone therapy

A number of randomised controlled trials and meta-analyses have been conducted on two particular interventions used clinically for PTB prevention: progesterone therapy and cervical cerclage ^28^. Despite the controversy over the effectiveness of these interventions in recent years, some clarity is emerging. Jarde *et al.*, in a recently published network meta-analysis, concluded that progesterone was more effective than cerclage for primary prevention of PTB in high-risk women with a singleton pregnancy ^29^. This is consistent with a recent patient-level meta-analysis, which concluded that cervical cerclage lacked demonstrable efficacy in women with a shortened cervix (<25 mm) ^30^, although this may depend on previous PTB status. On the other hand, progesterone therapy was found to significantly decrease rates of PTB at <34 weeks (OR 0.44) and <37 weeks (OR 0.58) and neonatal death (OR 0.50) ^29^. Romero *et al.* had earlier come to similar conclusions following their own re-analysis of published trials, supporting previous analyses of benefits and risks ^31^. Both of these meta-analyses included the OPPTIMUM trial ^32^, which failed to find a significant benefit of progesterone, although it reported some favourable trends. Importantly, the longer-term benefits of progesterone treatment to the child still remain to be proven ^32– 34^, and some authors have raised concerns regarding adverse effects on the developing brain ^35, 36^. This remains a major area of clinical uncertainty in need of solid, unambiguous data.

Progesterone’s effectiveness in women with a multiple pregnancy appears much less significant than in singleton pregnancies ^37– 40^, although the evidence that it lacks efficacy in this group ^41^ has recently been challenged ^42^; consensus on this topic remains elusive. The findings of several studies, including a recently published large trial, suggests that vaginal progesterone is more effective than intramuscular 17α-hydroxyprogesterone (17-OHP), although this may depend on various risk factors and aetiology ^37, 38, 43– 47^. Vaginal progesterone is certainly a much cheaper option. There is some evidence of increased risk of developing gestational diabetes with 17-OHP treatment ^43^.

One limitation of progesterone therapy is that it is applicable to only a small percentage of pregnant women (primarily those with a shortened cervix and those with a history of previous sPTB), so its net benefits on a population basis are limited ^4, 42, 48^. Nevertheless, several studies have now confirmed the feasibility of conducting population-based cervical screening programs ^28, 34, 49, 50^ and have concluded that the cost-effectiveness of such programs combined with progesterone therapy is favourable ^49, 51– 54^, although they would clearly be improved if the effectiveness of interventions could be increased ^55^.

We have some insight into how the effectiveness and response rates to progesterone therapy could be improved. Several lines of evidence suggest that inflammation in the cervix is required for effective vaginal progesterone treatment, which appears to work at least in part through an anti-inflammatory mechanism ^45, 48, 56– 58^. Markers of cervical inflammation could, therefore, be employed to target therapy to those at highest risk ^59, 60^. Alternatively, maternal blood-based tests ^61^ may be useful in identifying responders and non-responders as well as women with systemic inflammatory activation ^62– 64^.

### Cervical length screening

Measurement of the length of the cervix in mid-pregnancy using transvaginal ultrasound has been shown to be able to predict PTB with clinically useful reliability ^65– 68^. For PTB prediction, the sensitivity of a cervical length of <25 mm in women with a singleton gestation (no prior PTB) is ~40%, with a negative predictive value of 97% ^68, 69^; in women with a prior PTB, the sensitivity approaches 70% ^69^. The risk of PTB increases as cervical length decreases; in women with a cervix length of <15 mm the risk of sPTB approaches 50% ^68^.

A limitation of screening for shortened cervix is its low prevalence, ranging from 0.9 to 2.3% depending on the population and the cut-off employed (<15, 20, or 25 mm) ^54, 70^. It has been calculated that screening 10,000 asymptomatic pregnancies followed by progesterone therapy would likely prevent only 60 cases of PTB ^71^ and 16 deliveries at <33 weeks ^22^. Regions where PTB rates are low are likely to see even less benefit of screening and treatment. Nevertheless, given the potential mortality and morbidity of PTB, an investment in preventing this number of cases may indeed be warranted.

Some new sonographic approaches to cervical screening appear promising, however. Dziadosz *et al.* recently described the predictive performance of uterocervical angle measurement at 16–23 weeks’ gestation ^72^ They reported that an angle of ≥95° was significantly associated with sPTB at <37 weeks, with a sensitivity of 80% and a negative predictive value of 95%, while an angle of ≥105° predicted sPTB at <34 weeks (sensitivity 81%; negative predictive value 99%). This considerably outperformed standard transvaginal cervical length measurement.

Several groups have investigated the use of cervical ultrasound elastography to predict sPTB with and without cervical length assessment ^73, 74^. Two different approaches for quantitative determination of the physical properties of the pregnant cervix have been developed: strain elastography and shear wave elastography ^73^. In a small pilot study, a combination of strain elastography ratio and cervical length measurement was reported to greatly increase predictive performance, achieving an AUC of 0.88 ^75^. These advances open up the possibility of significant improvements in risk prediction and also response to treatment. However, they are still likely to predict only a small fraction of women who deliver preterm.

### PTB prevention programs and specialist clinics

A number of dedicated PTB prevention clinics have been established and evaluated over recent years. The first evaluation of such a program was the West Los Angeles PTB Prevention Project in the 1990s, which involved eight prenatal county clinics in California ^76^. The intervention, which included maternal education and increased clinic attendances, was reported to have achieved a significant 19% reduction in the PTB rate. More recent PTB prevention clinics have employed additional diagnostics and therapeutic interventions, including assessment of vaginal microbiology, fibronectin testing, ultrasound detection of shortened cervix, antibiotic use, progesterone therapy, cervical cerclage, and Arabin cervical pessaries. Most clinics treat women with a history of PTB or recurrent mid-pregnancy loss, previous PPROM, or previous loop excision or cone biopsy of the cervix. Manuck *et al*. found that in women who attended a dedicated PTB prevention clinic, recurrent PTB was considerably reduced (48.6% versus 63.4%) ^77^, as was the rate of composite neonatal morbidity (5.7% versus 16.3%) ^76^. The clinic consisted of three standardised clinic attendances, with routine administration of intramuscular 17-OHP and sonographic measurement of cervical length.

A UK survey of 23 dedicated PTB prevention clinics revealed considerable heterogeneity in protocols and practices, recommending improved networking, coordination, and consistency in referral criteria ^78^. PTB prevention clinics have now become an accepted part of antenatal care in many countries, making a systematic evaluation of their effectiveness very difficult ^79^. However, a recent review of the effectiveness of PTB prevention clinics found three trials with suitable data for analysis; a very modest, non-significant reduction in PTBs was found in the treatment group compared with controls (RR 0.87, 95% CI: 0.69 to 1.08) ^79^. There is clearly room for improvement.

Employing a more expansive strategy, the Western Australian PTB Prevention Initiative (WA-PTBPI) was recently established with the intention of safely lowering the rates of PTB in an entire state over a 5-year period ^22^. The initiative (badged “ *thewholeninemonths*”) is a multidisciplinary, multidimensional approach consisting of an outreach/educational program, a cervical length screening program, a public health/social media program, and a high-risk clinic located at the State’s obstetric tertiary referral centre ^80^. The key interventions are outlined in Table 1 ^80^. A recent analysis of the effectiveness of the initiative after the first 18 months of operation revealed a reduction in overall PTB (in singleton pregnancies) in the state by around 8% compared to pre-intervention rates (from 7.5 to 6.9%) ^80^. This reflects a state-wide reduction of approximately 200 preterm births, including more than 40 in the <32-week gestational age group. The rate of late PTB appeared to have decreased rapidly after the commencement of the initiative, suggesting an effect of educational programs aiming to discourage practitioners and women from unnecessary early intervention. The 28–31-week category had a more belated reduction, possibly reflecting benefits from the use of cervix length screening and vaginal progesterone administration ^80^.

**Table 1.** Key interventions of the Western Australian Preterm Birth Prevention Initiative *

1. Measurement of cervix length will be conducted routinely at 18–20 weeks’ gestation. In those cases in which the cervix can be imaged clearly on a transabdominal scan, a closed length from internal to external os of ≥35 mm is adequate. In all other cases, transvaginal scanning with an empty bladder is required at which a closed cervix length measured by this route of ≤25 mm is considered shortened.
2. Natural vaginal progesterone 200 mg pessary should be administered nightly for any case in which the cervix has been found on ultrasound imaging to be shortened between 16 and 24 weeks’ gestation. Treatment is to continue until 36 weeks’ gestation. In cases in which the cervix length is <10 mm on transvaginal imaging, management can include cervical cerclage, vaginal progesterone, or both.
3. Natural vaginal progesterone 200 mg pessaries for all cases in which there is a history of spontaneous preterm birth (with or without preterm pre-labour rupture of membranes) between 20 and 34 weeks’ gestation are to be used each night from 16 to 36 weeks’ gestation.
4. No pregnancy is to be ended prior to ≥38 weeks’ gestation unless there is a medical or obstetric justification.
5. Women who smoke should be identified and offered counselling though one of the well-established Quitline services offered through the Western Australian Department of Health.
6. A new dedicated and multidisciplinary preterm birth prevention clinic will be established at the tertiary-level centre for referral of high- risk cases. Typically, a management plan is developed and the woman is referred back to her referring practitioner when the high-risk period is concluded. Maternal-fetal medicine specialists, ultrasound imaging facilities for cervix length measurement, and mental health care and midwifery services are available within the clinic.

*Adapted from Newnham *et al*. ^80^

### Treatment and prevention of ascending intrauterine infection-related preterm birth

Ascending intrauterine infection is the major cause of early PTB and an important preventable cause for all PTBs ^1, 81, 82^. Preterm deliveries driven by infection are more likely to be associated with a) severe chorioamnionitis and funisitis, b) unresponsiveness to tocolysis, c) fetal inflammatory response syndrome (FIRS), and d) poorer neonatal outcomes ^1, 83– 85^. Some of the bacteria that regularly cause infection-driven PTB are common bacteria frequently found in the reproductive tract of pregnant women, but some are found in cases of abnormal vaginal microbiota (e.g. bacterial vaginosis [BV]) and/or are associated with reproductive tract infections ^86– 88^.

*Ureaplasma* spp. are the most common microorganisms isolated from the fetal membranes and amniotic cavity of cases of sPTB ^84, 85, 89– 91^. Vaginal colonisation rates of *Ureaplasma* spp. in pregnant women range from 35–90% ^92^. Overwhelming evidence shows a clear link between *Ureaplasma* spp. colonisation, a vigorous inflammatory response, preterm delivery, and adverse neonatal outcomes ^93– 97^. Two species of *Ureaplasma* are known to colonise the human vagina, *Ureaplasma parvum* and *Ureaplasma urealyticum* ^92^. We recently reported that 40% of pregnant women are colonised with *U. parvum*, while only 11% were positive for *U. urealyticum* ^98^. Importantly, *U. parvum* was detected in 77% of women who went on to have a sPTB compared to 36% in those who delivered at term. Even more significantly, *U. parvum* genotype SV6 was 3.6-fold more common in preterm deliveries than those at term, being detected in 54% of all sPTBs ^98^.

The identification of women at risk of an infection-related preterm delivery is far from simple and to date has relied predominantly on the diagnosis of BV for the recruitment of women to trials of prophylactic antibiotic administration for PTB prevention. However, BV is a less-than-optimal diagnostic criterion for risk prediction and trial inclusion. BV has been shown to be predictive of increased risk of PTB in populations with African ethnicities but is a relatively weak risk predictor in Caucasian populations (OR <2) ^99– 102^ with a low prevalence rate (<10%) ^99^. The diagnosis of BV fails to identify many women with vaginal dysbiosis who do not have BV symptoms but who may also be at high risk of infection-associated PTB. Most importantly, BV diagnosis does not take into account *Ureaplasma* spp. colonisation status or allow the classification of *Ureaplasma*-positive women to either high or low risk. A diagnostic test based on the presence of *U. parvum* SV6 and other bacteria is currently under development in our laboratory; in preliminary studies, it considerably outperforms BV as a risk marker and, moreover, identifies women who would benefit from antibiotic therapy and eradication of *U. parvum* infection.

Although several meta-analyses have concluded that antibiotic treatment of BV does not prevent PTB or improve neonatal outcomes ^103, 104^, a meta-analysis of trials of clindamycin treatment of women with BV prior to 22 weeks’ gestation showed a significant reduction in PTB at ≤37 weeks’ gestation plus a reduction in the incidence of late miscarriage ^105^. We have shown that *Ureaplasma* spp. colonisation of amniotic fluid occurs primarily after 20 weeks of pregnancy ^106^. Importantly, the antibiotics commonly used to treat BV (e.g. clindamycin) show poor activity against *Ureaplasma* spp., with evidence of significant and growing antibiotic resistance ^107– 109^. We have recently demonstrated the superiority of a new antibiotic called solithromycin, a fourth-generation macrolide developed to overcome macrolide resistance ^110^. It is highly potent against *Ureaplasma* and *Mycoplasma* species ^111^ plus is effective against all of the bacteria known to cause intra-amniotic infection. Furthermore, it is capable of crossing the placenta and treating the fetus ^110^. Solithromycin has not yet been approved for sale, however, and its safety has to be tested before its antenatal applications can be evaluated in clinical trials.

Conventional treatment of BV results in relatively high recurrence and relapse rates ^112– 114^. Probiotics (both vaginal and oral) have been shown to enhance the effectiveness of treatment of BV and candidiasis and markedly lower rates of recurrence ^113^. Probiotics are able to restore microbial homeostasis and exclude colonisation by pathogens ^112, 115^. It is likely, therefore, that probiotics administered after antibiotic therapy are likely to enhance treatment efficacy and reduce PTB rates; data from large randomised controlled trials to support this expectation are needed.

There is convincing evidence that screening for vaginal infections followed by antimicrobial treatment can significantly reduce the rates of PTB and improve perinatal outcomes. Kiss *et al.* recruited over 4,400 women in Vienna with singleton pregnancies ≤20 weeks of gestation ^116^. Women were screened for BV and presence of *Candida* spp. and if positive received antimicrobial treatment (clindamycin or clotrimazole as appropriate for six days), repeated if necessary after re-screening at 24–27 weeks. Treatment resulted in significant reductions in sPTB at ≤37 weeks (43% reduction) and miscarriage (64% reduction). In women who screened positive, the overall sPTB rate dropped from 7.0 to 2.9% with treatment; in women with BV, the PTB rate decreased by 38% (5.5 to 3.3%) and in women with *Candida* spp. it dropped by 66% (7.7 to 2.6%) ^116^. The magnitude of the effects observed is remarkable compared to the results of other interventions, particularly bearing in mind the poor risk prediction performance of the screening test (RR 1.3). The program was highly cost-effective ^117^, with estimated costs amounting to only 7% of the direct costs saved as a result of the reductions in prematurity (cost:benefit ratio 1:14). As a result of the success of this study, a voluntary antenatal infection “screen and treat” program was introduced in Vienna, offered to women with high risk of PTB due to obstetric risk factors. Recurrent infections were retreated and women were given probiotics after treatment to prevent BV recurrence ^118, 119^. Remarkably, the results actually exceeded the benefits of the original trial, proving that the benefits could be achieved in a routine clinical setting. Increased obstetric care and reassurance to clinic patients may have been a significant contributor to improved outcomes, independent of any direct interventions.

### Inflammation-associated preterm birth

Intra-amniotic infection and inflammation are key drivers of PTB and neonatal morbidity, particularly in infants delivered at ≤34 weeks’ gestation ^1, 17^. Several strategies have been explored to mitigate the effects of inflammation in the neonate ^120^; however, few interventions have been developed and evaluated for use prior to delivery, when the greatest therapeutic benefits exist ^17, 121^.

By conservative estimates, around 40% of all PTBs are caused by sterile or infection-related IUI, with rates ranging from ~90% of births at 21–24 weeks’ gestation to ~10% of deliveries at term ^1, 84^. IUI is commonly associated with PPROM, chorioamnionitis, and funisitis ^84^. Severe IUI is associated with FIRS as well as maternal, fetal, and newborn sepsis ^84^. In addition to maternal morbidity, IUI increases the risk of major fetal and newborn morbidity ^1^. Fetal inflammation leads to haematologic, endocrine, cardiac, renal, immune, and pulmonary abnormalities plus damage to the central nervous system, thymus, gut, and skin, with increased risk of developmental/behavioural abnormalities ^122, 123^.

The immature fetus may be exposed to inflammation as a result of microbial-driven chorioamnionitis, transplacental viral exposure, or sterile inflammatory insults such as danger-associated molecular patterns (DAMPs), oxidative stress and reactive oxygen species, maternal allograft rejection, uterine distension, senescence, or ischemia/hypoxia ^17, 84^. Antibiotics may be effective in eradicating infections but cannot treat sterile inflammation ^124^. To improve neonatal outcomes, anti-inflammatory interventions are needed to complement antibiotic therapy ^121^.

A variety of anti-inflammatory pharmacological approaches have been explored to protect against the adverse effects of inflammation in pregnancy ^120, 124, 125^. The therapeutic strategies include a) long-term prophylactic administration to limit excessive inflammation in at-risk women and b) acute administration to women in preterm labour to mitigate the effects of inflammation on the fetus. Pharmacological considerations for these two scenarios are different.

A series of studies exploring the use of cytokine-suppressive anti-inflammatory drugs (CSAIDs) have shown pharmacological efficacy in animal and human *ex vivo* models ^124^, demonstrating the ability to block inflammation in human preterm fetal membranes delivered following spontaneous preterm labour ^126^ and LPS-driven inflammation in a sheep model via intra-amniotic injection ^125^. The effects of these drugs on fetal inflammation following maternal administration have not yet been evaluated, however, and their use in the clinic is still some way off.

The compound (+)-naloxone is a potent TLR4 signalling antagonist and a non-opioid isomer of the widely used opioid receptor antagonist (-)-naloxone ^127^. (+)-naloxone suppresses immune NF-κB activation and cytokine biosynthesis ^128– 130^, protecting against sepsis in animal models ^131, 132^. (+)-naloxone administered in late gestation delays the timing of birth by 16 hours ^133^ and alleviates fetal demise after intraperitoneal LPS administration ^134^. In a model of *Escherichia coli*-induced IUI, (+)-naloxone protected pups from PTB and perinatal death ^134^ and suppressed cytokine expression in fetal membranes, placenta, uterine myometrium, and decidua ^134^. Importantly, (+)-naloxone had no adverse effects on fetal or neonatal development, and the history of (-)-naloxone use in pregnancy suggests that the drug is safe and without significant risks.

An alternative approach that has been tested is to block the actions of key cytokines such as IL-1, which plays a pivotal role in uterine and placental inflammation and also in mediating the adverse effects of inflammation in the fetal brain and other organs ^135^. Studies using the commercially available antagonist Kineret have been overshadowed by a new, allosteric peptide IL-1R antagonist known as rytvela ^136^. Rytvela treatment in pregnant mice improved fetal and neonatal outcomes following exposure to either IL-1β or LPS ^136– 138^, blocked uterine/placental/intra-amniotic inflammation, and prevented myometrial activation, PTB, fetal demise, and inflammation-related morbidities; rytvela-exposed pups exhibited overtly normal growth and development ^137, 138^. In all studies, rytvela equalled or outperformed Kineret, despite being used at lower doses. This exciting pharmacological advance is undergoing further preclinical evaluation prior to clinical trials.

Statins have also been proposed to have a role in mitigating the negative effects of inflammation in pregnancy ^139^. Studies in the LPS mouse model have shown that statin administration prevents cervical remodelling, myometrial contractility, and PTB ^140^ and reduces cytokine expression in the uterus, cervix, serum, and amniotic fluid ^141^. Similar effects have been shown in human fetal membranes ^142^. Several large prospective studies are underway to confirm reports ^143, 144^ that statin administration is safe in pregnancy.

## Concluding comments

The PTB prevention landscape has altered significantly in the last decade or so, with the mainstream introduction of dedicated PTB prevention clinics, transvaginal ultrasound cervical length screening programs, and progesterone administration to women at high risk ^22^. However, the gains achieved by these initiatives are modest at best, and there is significant scope to improve the effectiveness and targeting of therapies and the accuracy of our risk assessment strategies. To this end, several promising developments that have the potential to significantly lower PTB rates have been described. In going forward, we must be mindful that the primary goal is to improve outcomes for the infant in both the short and the long term. This requires trialists to employ a consistent set of endpoints ^145– 147^ that include the assessment of a range of neonatal/paediatric respiratory and neurodevelopmental parameters ^32, 34^.

One also has to be realistic in terms of what can be achieved. No intervention is 100% effective, and a significant proportion of PTBs are the result of unidentified causes and are completely unpredictable by current methods ^22^. Furthermore, as a syndrome, PTB is the result of many different aetiologies and as such may require multiple interventions targeted to different subgroups based on risk stratification and prognostic profiling. Nevertheless, as the research reviewed in this article highlights, there are a number of avenues where it appears that significant progress can be made and further advances are highly likely; the opportunities for short-to-medium gains are plentiful.
